# Current State of the First COVID-19 Vaccines

**DOI:** 10.3390/vaccines9010030

**Published:** 2021-01-08

**Authors:** Birgit M. Prüβ

**Affiliations:** Department of Microbiological Sciences, North Dakota State University, Fargo, ND 58104, USA; Birgit.Pruess@ndsu.edu; Tel.: +1-701-231-7848

**Keywords:** SARS CoV-2, COVID-19, vaccine development, phase III trial

## Abstract

SARS CoV-2 and its associated disease COVID-19 has devastated the world during 2020. Masks and social distancing could be efficient if done by large proportions of the population, but pandemic fatigue has decreased their efficacy. Economic shut downs come with large price tags and cannot be a long term solution either. The announcements by three vaccine manufacturers in November that their vaccines are 90% or more effective has given hope to at least those in the population who plan to get vaccinated as soon as a scientifically and medically sound vaccine becomes available. This review summarizes the underlying design strategies and current status of development of the nine vaccines that were in phase III trial on 8 November 2020. Contracts between vaccine manufacturing companies and governments aim at distributing the vaccine to a large part of the world population. Questions remain how the temperature sensitive mRNA vaccines will be transported and/or stored and how vaccination will be prioritized within each country. Additionally, current contracts do not cover all countries, with a serious gap in Africa and South America. The second part of this review will detail current distribution plans and remaining challenges with vaccine accessibility and acceptance.

## 1. Introduction

Since the first cases of COVID-19 in China about a year ago [1], the novel coronavirus causing severe acute respiratory syndrome (SARS) Cov-2 has by now caused 84,233,579 confirmed cases and 1,843,293 confirmed deaths (World Health Organization, WHO, www.who.int/data; 5 January 2020). This novel coronavirus is different from previous coronaviruses that infect humans in that disease progression is more difficult to predict and also more diverse. Early symptoms are similar to SARS CoV and MERS [2], but then SARS CoV-2 is capable of spreading to many different organs in the human host thanks in part to using the angiotensin converting enzyme-2 receptor (ACE-2) that is abundant in many organs [3,4]. In severe cases, acute respiratory distress symptom [5] and cytokine storms [6] can lead to death of the patient. In addition to respiratory and cardiovascular symptoms, a range of neurological symptoms can occur [7] and altogether, COVID-19 is much more than a respiratory disease [8]. Likewise, long-term health consequences of infection with SARS CoV-2 include respiratory [9] and immunological complications [10].

Early intervention attempts included travel bans and economic lock down [11,12]. Due to the high economic cost, these are really only useful to buy time for the development of drugs and vaccines [13] and not considered long-term solutions. Current interventions includes testing for viral RNA and SARS CoV-2 specific antibodies, facial coverings, contact tracing, and isolation [14,15,16,17,18]. However, community spread together with pandemic fatigue (e.g., long-term stress response, including difficulty in abiding by restrictions such as wearing masks) have rendered some of these interventions less effective and the need for novel drugs and vaccines is more evident than ever. This author reviewed drugs that are targeted against the virus in a previous article [19]. In this new article, the current status of nine COVID-19 vaccines that were in phase III trial in November of 2020 will be summarized and compared. Current distribution plans are investigated.

## 2. Current Status of Vaccine Development

A quick search on 26 November 2020 on ClinicalTrials.gov (https://clinicaltrials.gov), using COVID vaccine as search term yielded 141 studies. Of these, 43 were phase III trials. This chapter details information about nine SARS CoV-2 vaccines that were developed in the EU, US, Russia, China, and Australia and were in phase III trials on 8 November 2020. Some of these have been included in a recent review on the design of SARS CoV-2 vaccines [20]. Basic information is provided on the design of the vaccine, which is either mRNA, adenovirus vector, protein subunit, inactivated SARS CoV-2, or a live attenuated strain of *Mycobacterium bovis*. Phase I and II trials are completed for six of the nine vaccines and the outcomes are published. Phase III trial data are published for two vaccines and ongoing for the others. Table 1 summarizes the nine vaccines. Note that there are many additional vaccines (e.idem by Arcturus, Merck) that are expected to go into phase III trials during 2021.

### 2.1. BioNTech/Pfizer Vaccine (Germany/US)

A vaccine that has caused a large amount of publicity in November is the one that was developed by the German company BioNTech and the US pharmaceutical giant Pfizer. BNT162b1 and BNT162b2 are examples of mRNA vaccines, where a synthetic mRNA is injected and translated quickly into protein by the host. mRNA is a reasonably new class of drugs that deliver genetic information [32]; the technology was originally developed for cancer immunotherapies [33]. Treatment based on mRNA is considered safe because the RNA is transiently expressed, metabolized quickly and does not integrate into the host genome [34]. Lately, modifications of the mRNA with 1-methylpseudouridine has resulted in a longer lived antibody response, while packaging the mRNA into liquid nanoparticles serves as protection from degradation [35]. An additional strength of this technology is the ability to quickly produce large amounts off vaccine against novel pathogens, which is a requirement for a worldwide distribution of the vaccine [32].

The development of BNT162b1 and BNT162b2 started in Germany at BioNTech. BNT162b1 encodes the trimerized (by addition of T4 fibritin folding domain) receptor binding domain (RBD) of the spike protein. BNT162b2 encodes the prefusion stabilized membrane-anchored full length spike protein, modified by two proline mutations to maintain prefusion conformation. The spike protein is considered a prime target for virus neutralizing antibodies [36]. A cationic lipid nanoparticle consisting of ionizable amino lipid, phospholipid, cholesterol and a polyethylene glycol-lipid was described earlier and aids in the delivery of the mRNA into the cytosol [37]. BNT162b1 elicited a good RBD-binding IgG antibody and T cell (RBD-specific CD8(+) and CD4(+)) response [38]. The immunological response to BNT162b2 was similar, but this vaccine was associated with less systemic reactogenicity (e.g., fever, fatigue, headache, grade 4 severe reactions) in particularly in the 65–85 year age group [39]. Phase I/II trials are ongoing in Germany for BNT162b1/BNT162b2 (NCT04380701) and China for BNT162b1 (NCT04523571). NCT04368728 is a phase I/II/III trial that tests BNT162b1 and BNT162b2 with 43,448 participants. In several groups, participants between the age of ≥12 years up to 85 years were tested, two doses were given by intramuscular injection. Local and systemic reactions were monitored, SARS-CoV-2 serum neutralizing antibodies were measured, as well as anti-RBD binding antibody levels. The phase II component of this trial was published [21]. Two doses of BNT162b1, administered to 45 adults between the ages of 18 and 55 demonstrated that the vaccine resulted in robust immunogenicity. Seven days after a second dose of 30 µg of BNT162b1, given 21 days after the first dose, RBD-binding IgG increased to 27,872 U/mL and persisted for another week. In comparison, human convalescent sera only contained 602 U/mL of RDB-binding IgG. Likewise, SARS CoV-2 neutralizing titers reached 437 at 14 days after the second dose of 30 µg, as compared to 94 from human convalescent sera. Symptoms included mild to moderate pain at the injection site and mild to moderate fatigue and headache. Most these reactions improved after two days [21]. The phase III trial component of NCT04368728 identified eight cases of COVID-19 in participants that had received BNT162b2 and 162 cases among the participants that had received the placebo [22]. This was considered 95% effectiveness. BioNTech/Pfizer have recently received emergency use authorization (EUA) from the FDA [40] and approval by the UK [41]. Pfizer has contracts with the US, EU, UK, Japan, Canada, and Australia. Rollout of the vaccine has started [42].

### 2.2. Moderna Vaccine (US)

A vaccine that builds on the same principle of using mRNA to express protein is mRNA-1273 by the US company Moderna. mRNA-1273 encodes the SARS CoV-2 glycoprotein with transmembrane anchor and intact S1 (binding)-S2 (fusion) cleavage site; two proline substitutions at positions 986 and 987 stabilize the spike protein in the prefusion conformation to increase immunogenicity [43]. To increase biostability, uridine is replaced by N1-methyl-pseudouridine [44]. The mRNA is delivered into the human cells by a nanoparticle, consisting of four lipids. The precise composition of the nanoparticle is unknown, however previous vaccines by Moderna use ionizable lipid, 1,2-distearoyl-*sn*-glycero-3-phosphocholine, cholesterol, and polyethylene glycol-lipid [45,46]. mRNA-1273 induces potent antibody and T-cell (CD8(+)) responses towards SARS CoV-2 and protects mice from infection with SARS CoV-2 [43]. An interesting study tested the efficacy of the vaccine in non-human primates after upper- and lower-airway challenge with SARS-CoV-2 [47]. Vaccination resulted in a robust virus neutralizing activity and protection of the upper and lower airways. It did not cause any pathologic changes in the animal lungs. A phase I trial (NCT04283461) tested the vaccine with (i) 45 healthy human adults between the age of 18 and 55 and (ii) 40 older adults [23,24]. Two doses of 25 or 100 µg were given intramuscular at 28 days apart. Antibody and virus neutralization responses were appropriate in both age groups, and no safety concerns were observed. The phase III trial NCT04470427 tests the vaccine efficacy by injecting 100 µg of mRNA-1273 at days 1 and 29 and measuring first occurrence of COVID-19 at 14 days after the second dose, adverse events, local and systemic reactions, and immunological response. The trial involves 30,000 participants, of which 37% are from minority communities and 42% from the high risk category. Preliminary data were revealed by several main stream media that indicated a 94.5% effectiveness against infection with COVID-19. Moderna has received EAU from the FDA [48]. The US Department of Health and Human Services (HHS; http://www.hhs.gov) has a contract with Moderna to secure the first 100 million doses. Roll out in the US started during the second half of December. Other countries that Moderna has contracts with include Canada, Japan, the EU, and Switzerland.

### 2.3. Oxford/Astra Zeneca Vaccine (UK/US)

The design for the AZD1222 (ChAdOx1 nCOV-19) vaccine by the University of Oxford in the UK and Astra Zeneca in Cambridge, UK is fundamentally different from the mRNA vaccines. Instead of using mRNA and a nanoparticle, this vaccine is based on a replication-deficient chimpanzee adenovirus. Viral vectors have been used for the development of vaccines for several decades and are characterized by a strong CD4+ and CD8+ response even in the absence of an adjuvant, which makes them a suitable vaccine vector for pathogenic viruses that elicit a strong cellular immune response [49]. In addition to human adenoviruses (e.g., Ad5, Ad26), simian adenoviruses have been used because of the reduced seroprevalence in the human population, leading to reduced pre-existing immunity [50]. AZD1222 is based on the ChAdOx1 chimpanzee adenovirus, which carries deletions in the early genes E1 and E3 that are intended to inhibit replication and permitting for the integration of large pieces of genetic information. The integrated gene encodes the full length spike protein in its glycosylated form and includes a tissue plasminogen activator leader sequence [25]. A single dose induced antibody and T cell responses in mice and pigs, while a second immunization increased virus neutralizing titers in pigs [51]. In macaques, the vaccine induced a balanced humoral and cell-mediated immune response and reduced viral load; pneumonia was reduced in vaccinated and consecutively infected animals [52]. A phase I/II clinical trial (NCT04324606) with 1077 participants demonstrated that adverse effects of the vaccine, administered intramuscularly, were mild to moderate and included fever, headaches, body pain and malaise that could be suppressed with paracetamol. The T cell response peaked on day 14, the IgG antibody response at day 28. The latter was further improved by a second immunization. Neutralization response occurred in 91% of the participants after one immunization and 100% after the second boost [25]. The phase II component from the phase II/III trial NCT04400838 was published and contained data from 560 participants [26]. Adverse effects were similar to the ones reported previously, but less severe in older participants than in young ones. While 13 serious adverse events occurred, these were considered unrelated to the vaccine. The responses for binding and neutralizing antibodies, as well as the T cell response were in the appropriate range. The phase III component from this trial tests the vaccine on 12,390 participants. A recent publication provides an interim efficacy analysis from a total of 11,636 participants that were enrolled in four trials (NCT04324606, phase I/II; NCT04400838, phase II/III; NCT04444674, phase I/II, and ISRCTN89951424) in South Africa, Brazil, and the UK [27]. The efficacy of preventing COVID-19 in a test group that had received two full doses of the vaccine was 62.1%, whereas the efficacy in a test group that had received a lower first dose followed by a full second dose was 90%. Across all participants, the efficacy was 70.4%. Another phase III trial (NCT04516746) tests the efficacy of the vaccine on 40,051 participants in two doses of 5 × 10^10^ virus particles, administered four weeks apart. Measurable outcomes are the prevention of COVID-19, adverse reactions, and antibody and neutralization responses. Astra Zeneca has contracts with the US, EU, UK (just approved), India, China, Thailand, The Philippines, and Australia.

### 2.4. Janssen Vaccine (The Netherlands/US)

The Ad26.COV2.S vaccine by the Janssen Vaccines and Prevention BV subsidiary of Johnson and Johnson is also based on an adenovirus vector, the human adenovirus Ad26 [53]. Ad26.COV2.S expresses the full length spike protein, stabilized by furin cleavage site mutations and two consecutive proline stabilizing mutations in the hinge region. It contains the wild-type signal peptide. This vaccine exhibits potent neutralizing immunity [54]. A single immunization with this vaccine administered intranasally or intratracheally elicited binding and neutralizing antibody responses and reduced the occurrence of COVID-19 in hamsters [55] and rhesus macaques [56]. Two phase I/II trials in the US (NCT04436276) and Japan (NCT04509947) assess safety, reactogenicity, and immunogenicity after intramuscular administration of the vaccine in one or two doses. A phase III trial (NCT04505722) tests the efficacy of the vaccine in 60,000 participants after a single intramuscular dose of 5 × 10^10^ virus particles. Measurable outcomes will be the occurrence of COVID-19, viral load by qPCR, systemic and local adverse effects, and binding/neutralizing antibody titers. This trial was briefly interrupted because of unexplained illness in a participant [57]. The phase III trial NCT04614948 with 30,000 participants places emphasis on the molecular confirmation of COVID-19 and co-morbidities. The EU has a contract with Johnson and Johnson for 400 million doses, the US for 100 million doses.

### 2.5. Novavax Vaccine (US)

The NVX-CoV2373 vaccine by Novavax (Gaithersburg, MA, US) is the full length SARS CoV-2 spike protein subunit in its glycosylated form. Mutations at the S1/S2 cleavage site protect the spike protein against proteolytic degradation; proline substitutions help maintaining the prefusion conformation [28]. The conformation of this synthetic spike protein is almost identical to the natural protein with a small difference in the S1 subunit and normal interactions between the spike trimers [58]. To increase immunogenicity, the saponin-based adjuvant Matrix-M is included in the vaccine; spike protein subunit and adjuvant are mixed prior to injection [59]. NVX-CoV2373 was first tested in macaques (*Macaca fascicularis*) that were immunized with the vaccine and challenged with the native virus intranasally and intratracheally [60]. The monkeys were protected against COVID-19 and did not exhibit any symptoms of upper or lower airway infection or pulmonary disease. The phase I/II clinical trial with 131 adult and healthy humans (NCT04368988) tested the efficacy of two intramuscular injections, 21 days apart, in doses of 5 or 25 µg, with or without Matrix-M1 adjuvant. Adverse effects were mild to a maximum of mild fever for one day in one volunteer. The addition of the Matrix-M adjuvant increased the immune response, as measured by antibody and neutralization response and T helper 1 response, all of which were above those measured from convalescent serum [28]. The phase III trial NCT04611802 tests the efficacy of the vaccine on 30,000 participants in the US and Mexico in two doses of 5 μg SARS-CoV-2 rS and 50 μg Matrix-M1 adjuvant at 21 days apart. Measurable outcomes include the occurrence of COVID-19 after 28 to 750 days, first occurrence of a first PCR-positive test results, and immunological response, ACE-2 receptor binding inhibition, and adverse effects. Another phase III trial in the UK (NCT04583995) tests the efficacy of the vaccine on 15,000 participants. Measurable outcomes are the occurrence of different levels of COVID-19 and adverse effects. The Novavax vaccine is on the fast track for FDA approval. The HHS has a contract with Novavax for 100 million doses.

### 2.6. Sputnik V Vaccine (Russia)

The Russian vaccine Sputnik V (also known as Gam-Covid-Vac) was developed by the Gamaleya National Center of Epidemiology and Microbiology and is based on both of the human adenovirus vectors rAd26 and rAd5. This type of heterologous prime-boost vaccination is being done to overcome negative impacts of the immune response to components of a particular vector [61]. Both, rAd26-S and rAd5-S carry the SARS CoV-2 full length spike glycoprotein. The phase I trial NCT04436471 used one intramuscularly administered dose of either rAd26-S or rAd5-S and determined the safety of the vaccines. In the phase II trial NCT04437875, the first vaccination was done intramuscularly with rAD26-S, a second vaccination with rAd5-S after three weeks. Adverse effects included pain at the injection site, headache, and muscle pain. Binding and neutralizing antibodies were increased and the T-cell response for CD4+ and CD8+ were appropriate [29]. These initial good news were sufficient for the vaccine to get approved by the government on 11 August 2020, which made Russia the first country to register a COVID-19 vaccine. However, the approval was done before the phase III trial started, a decision that was questioned in multiple articles in high profile Journals [62,63,64]. The ongoing phase III trial NCT04530396 tests the efficacy of the vaccine on 40,000 participants with a day 1 intramuscular injection of 0.5 mL of rAd26-S and a day 21 dose of 0.5 mL of rAd5-S. Measurable outcomes are the development of COVID-19, severeness of Covid progression, antibodies, and cellular immunity, and adverse effects. Main stream media reported that three health care workers who had received Sputnik V tested positive for SARS CoV-2. An Indian pharmaceutical company, Hetero, is contracted to produce 100 million doses of this vaccine. Note that a second COVID-19 vaccine was approved by Russia on October 14, named EpiVacCorona. This vaccine was developed by the Vector State Research Centre of Virology and Biotechnology. A single phase I/II trial (NCT04527575) was retrieved for this vaccine that started on 26 August. This author was unable to retrieve a single peer-reviewed Journal article on EpiVacCorona or an ongoing phase III trial until 5 January 2020.

### 2.7. CanSino and Sinovac Vaccines (China)

Two vaccines are currently in trials in China. The Ad5-nCOV vaccine by Beijing Institute of Biotechnology (Beijing, China) and CanSino Biologics (Tianjin, China) is based on the human adenovirus Ad5. The Ad5 vector carries deletions in the E1 and E3 early genes, expresses the full length spike glycoprotein (GenBank accession number YP_009724390), and carries the plasminogen activator signal peptide gene [30]. Ad5-nCOV was first tested in mice and ferrets. Mucosal vaccination was successful at protecting mice completely from upper and lower respiratory tract infections and ferrets from infection of the upper respiratory tract [65]. The phase I trial NCT04313127 with 108 participants determined that administering one intramuscular dose of the vaccine is well tolerated and appropriate antibody and T-cell responses were noted after 14 and 28 days [30]. The phase II trial NCT04341389 determined 5 × 10^10^ virus particles as an effective dose for vaccination, as a dose of 1 × 10^11^ virus particles let to severe adverse effects in 9% of the participants [31]. The phase III trial NCT04540419 with 500 participants tests the effect of a single vaccination with 5 × 10^10^ virus particles on the occurrence of COVID-19, severeness of disease progression, immunogenicity, adverse effects, serum chemistry, and blood counts.

The Corona Vac vaccine by Sinovac Biotech in China is inactivated SARS CoV-2 virus and uses aluminium hydroxide as adjuvant. Three phase I/II trials (NCT04551547, NCT04383574, NCT04352608) on 552, 442, and 744 participants, respectively, test the immunogenicity and safety of the inactivated SARS-CoV-2 vaccine. Two doses are administered 14 or 28 days apart at doses of 300 SU/0.5 mL, 600 SU/0.5 mL, and 1200 SU/0.5 mL. The measurable outcomes are the titer of neutralizing antibodies and adverse effects. The phase III trial NCT04617483 evaluates the ‘non-inferiority of the commercial scale inactivated SARS-CoV-2 vaccine’ on 1040 participants with two doses of 600 SU/0.5 mL, administered 14 days apart. The measurable outcome is the titer of neutralizing antibodies and adverse effects. According to the state-owned Chinese pharmaceutical company Sinopharm, the inactivated SARS CoV-2 vaccine has already been administered to 1 million of people (The Guardian, US News, MSN). Additional phase III trials for this vaccine are being performed in Turkey (NCT04582344) and Indonesia (NCT04508075). A research paper published the protocol for a phase III trial (NCT04456595) in Brazil [66], the vaccine is administered intramuscularly to 13,060 participants in two doses at 14 days intervals.

### 2.8. BCG Vaccine (Australia)

BCG, developed by Murdoch’s Childrens Research Institute and Royal Children’s Hospital in Australia, is named after Bacille Calmette-Guérin and contains live attenuated *Mycobacterium bovis.* While the mechanism by which BCG protects humans from a range of infectious diseases is largely unknown, epigenetic reprogramming leading to trained immunity has been proposed as one possibility with IL-1β being a key player [67]. Based on 100 years of using BCG as a vaccine, BCG is considered safe. The principal of using BCG against COVID-19 has been proposed because of its effectiveness against tuberculosis [68] and questioned because of the non-specificity of the immune response [69]. First evidence that BCG might be effective against COVID-19 was obtained from a retroactive cohort study. It was determined that people who had received BCG within the past five years preceding the COVID-19 pandemic had a lower incidence of sickness with COVID-19 (adjusted odds ratio 0.58, *p* < 0.05) [70]. Additional evidence comes from an observational study that noticed that COVID-29 was less wide spread in countries that use child vaccination with BCG than in countries that do not [71]. However, whether vaccination with BCG can really protect humans from COVID-19 can only be determined by means of large randomized trials. The phase III trial NCT04327206 tests the vaccine on 10,078 participants that are administered 0.1 mL of intradermally. Each 0.1 mL vaccine contains between 200,000 and 800,000 colony forming units of BCG. Measurable outcomes include the occurrence of COVID-19 and severe COVID-19 by 12 month, work absenteeism, pneumonia, mechanical ventilation, mortality, local, and systemic adverse effects. Other countries that are currently testing this vaccine in phase III trials are the US (NTC04632537), Egypt (NCT0350931), Netherlands (NCT04328411), and South Africa (NCT04379336. A phase IV trial, NCT043485470 has just started in the US. Measurable outcomes of these trials are absence and hospitalization of healthcare workers, as well as incidence of COVID-19 infection.

## 3. What Is Up Next: Vaccine Effectiveness, Accessibility, and Distribution

**Figure 1:**
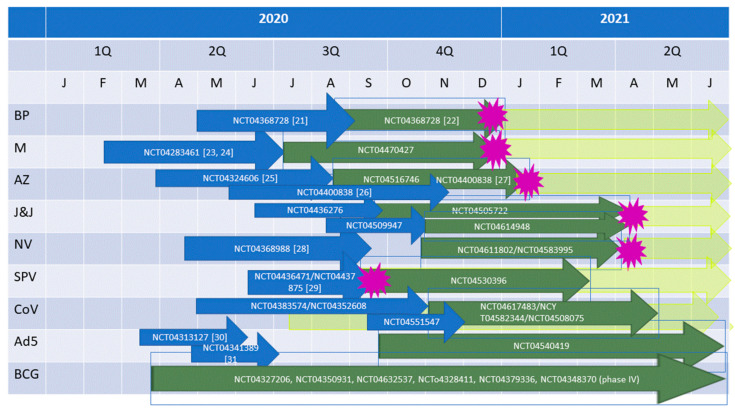
Time line of vaccine production and approval. BP, BioNTech/Pfizer; M, Moderna; AZ, Oxford/Astra Zeneca; J&J, Janssen/Johnson and Johnson; NV, Novavax; SPV, Sputnik V; CoV, CoronaVac; Ad5, Ad5-nCOV; BCG, Mycobacterium bovis. Blue arrows, phase I/II trials; green arrows, phase III trials; yellow arrows, roll out; purple star, (anticipated) approval date. Trial start dates were taken from http://www.clinicaltrials.gov. Approximate end dates of phase I/II trials are the publication dates. End points for unpublished trials a4re guesses by the author. For the last three vaccines, a guess for an approval date was not possible. Note that all start and end dates of trials, as well as approval dates are approximations.

After the comparative review of the nine phase III trial vaccines, this chapter will seek to answer questions as to the (i) first time availability of the vaccines, (ii) large scale production of the vaccines, (iii) distribution, (iv) transport and storage, and (v) long-term effectiveness. Questions that cannot be answered based on the current state of knowledge are posed. Figure 1 provides a time line of vaccine production and (anticipated) approval dates.

The announcements in November by BioNTech/Pfizer, Moderna, and Oxford/Astra Zeneca about their respective vaccines being ≥90% effective is certainly exciting. Of course, many questions remain open. General advantages of mRNA drugs are the simplicity of their production and their inexpensiveness [32]. Among the disadvantages are the inherent instability of mRNA, though some of that has been overcome with the described modifications [35]. The BioNTech/Pfizer vaccine needs to be stored at –80 °C, while the Moderna vaccine can be stored in a refrigerator short term and at –20 °C longer term. The Oxford/Astra Zeneca vaccine is refrigerator stable, production of a single dose costs only ~$3 (as opposed to up to $20 for the mRNA vaccines). For the BioNTech/Pfizer vaccine, it has been questioned whether the vaccine works in particular demographic groups and whether it stays effective beyond the two month of the current trial [72]. A real intriguing question is whether the vaccine can prevent transmission by COVID-19 patients that are asymptomatic [72]. The answers to these questions cannot be given with the current trial, but will require large scale vaccination and long-time observation. Of course, much of the same applies to the Moderna and Oxford/Astra Zeneca vaccines, though Moderna released data that the vaccine reduced severe COVID-19 [73], a distinction that had not been made by Pfizer. Astra Zeneca makes age distinctions among patients.

The BioNTech/Pfizer and Modera vaccines have been given EUA by the FDA, Pfizer has received approval of their vaccine in the UK and applied for approval in Germany, the Astra Zeneca vaccine has been approved in the UK. Astra Zeneca is applying for emergency approval for distribution worldwide. This includes 100 million doses for people in the UK. An important question that arises at this stage is the prioritization of the vaccine within the population of a given country. In the US, the CDC has published their prioritization and consideration guidelines [74] and recommends the vaccination of 17–20 million health care workers during the limited dose availability phase, and the vaccination of 60 to 80 million essential workers during the large number of doses availability phase, followed by people with high risk conditions and the elderly (>65 years). Racial/ethnic considerations are included in each of these groups. The CDC also announced fours states and a city (California, Florida, Minnesota, North Dakota, Philadelphia) for a pilot program [75]. The EU has contracts with Pfizer and Moderna, as well as other companies. The European Medicines Agency (EMA; http://www.ema.eu) is reviewing three vaccines (BioNTech, Moderna, Janssen) and expects roll out in 2021. EU member states will receive vaccines according to their population size, starting to vaccinate health care workers, people with high risk conditions, and elderly. The Joint Committee on Vaccination and Immunization (JCVI) in the UK recommends an age based prioritization of the vaccine [76]. The BioNTech/Pfizer vaccine was approved by the Medicines and Healthcare products Regulatory Agency (MHRA; Medicines and Healthcare products Regulatory Agency—GOV.UK (www.gov.uk)) in the UK on 2 December 2020 [41]. In Germany, the Paul-Ehrlich Institut (https://www.pei.de) is responsible for vaccine safety. The first phase of vaccination in Germay has a focus on health care workers and members of vulnerable groups.

While the western part of the world performs their phase III trials first and gives emergency use approval for the vaccines after that, Russia and China already vaccinated people before their phase III trials were started/completed. Russia was the first to register their vaccine and some 40,000 Russians were vaccinated after that. Several general media outlets reported that three of the doctors who got the vaccine got sick with COVID-19. A press release from 24 November 2020 (https://sputnikvaccine.com/newsroom/) promised the production of a billion of doses during 2021 at a production price of less than $10 and a need for two doses. Preliminary data from the phase III trial indicate an efficiency that is similar to the BioNTech/Pfizer and Moderna vaccines. A willingness was expressed to provide the vaccine to other countries who need it, though no countries were specified. In China, the phase III trial for the Sinovac vaccine just started in November and yet 1 million of people have already been vaccinated. The latest general media press releases point towards a quick immune response but with antibody levels that are lower than in recovered patients. The author or this article was not able to find information on how the Russian and Chinese vaccines are prioritized within the respective populations.

Looking at the countries that are producing the vaccines and the countries the companies are contracted with, there appears to be broad coverage within Europe, North America, Australia, as well as parts of Asia. There seems to be a gap, however, covering much of South America and Africa. Admittedly, the transmission of COVID-19 in Africa and South America (exception Brazil) started later and is still somewhat lower than in other parts of the world [77,78,79]. In fact, the coronavirus map by John’s Hopkins [80] lists the US, India, and Brazil first, followed by several European countries (including Russia) and shows a significant gap in Africa. Additionally, differences in reporting, difference in the age profile of the populations could be among the reasons for this discrepancy.

Of course, vaccine acceptance is an additional problem, which has been known before the COVID-19 pandemic [81,82] and has recently been fueled by conspiracy theories [83]. A formal survey of 13,426 people in 19 countries determined that 71.5% of the participants would be open to taking a COVID-19 vaccine with large variations within countries (China, 88%; Russia 55%) [84]. In the US, vaccine acceptance has been determined to be at 67%, but with large differences between demographics [85]. It seems like making the vaccine accessible to every world citizen, as well a convincing people that vaccines are not the enemy will be one of the major challenges the world will face in 2021 and every progress that will be made with vaccine development, phase III trial completion, and approval will be work in progress until the vaccine has been delivered to the people.

## 4. Conclusions

Altogether, the newly developed COVID-19 vaccines come with promise for a future that is brighter than 2020, but also with challenges. Two of the discussed vaccines received EUA by the FDA and one of them is expected to receive EUA status soon. The UK have approved several of the vaccines. Russia and China started vaccinating during the second half of 2020, other countries started in December of 2020. The year 2021 will show how these vaccines will be rolled out and whether the desired goal of controlling the COVID-19 pandemic will be accomplished.

## Figures and Tables

**Table 1:** Comparison of nine vaccines that are currently in phase III trials.

Vaccine	Institution	Country	Mechanism	Phase I/II Trials	Phase III
BNT162b1/ BNT162b2	BioNTech/ Pfizer	Germany/US	mRNA	NCT04380701 NCT04523571 NCT04368728 [21]	NCT04368728 [22]
mRNA-1273	Moderna	US	mRNA	NCT04283461 [23,24]	NCT04470427
AZD1222	University Oxford/ Astra Zeneca	UK	Adenovirus vector, chimpanzee	NCT04324606 [25] NCT04400838 [26]	NCT04400838 [27] NCT04516746
Ad26.COV2.S	Janssen/Johnson & Johnson	The Netherl./US	Adenovirus vector, Ad26	NCT04436276 NCT04509947	NCT04505722 NCT04614948
NVX-CoV2373	Novavax	US	Protein subunit	NCT04368988 [28]	NCT04611802 NCT04583995
Sputnik V (Gam-Covid-Vac)	Gamaleya Nat. Center of Epidem. and Microbiol.	Russia	Adenovirus vectors	NCT04436471 and NCT04437875 [29]	NCT04530396
CoronaVac	Sinovac Biotech	China	Inactivated SARS CoV-2	NCT04551547 NCT04383574 NCT04352608	NCT04617483 NCT04582344 NCT04508075
Ad5-nCOV	CanSino Biologics	China	Adenovirus vector	NCT04313127 [30] NCT04341389 [31]	NCT04540419
BCG vaccine	Murdoch’s Childrens Res. Inst./ Royal Children’s Hospital	Australia	Live attenuated *M. bovis*		NCT04327206 NCT04350931 NCT04632537 NCT04328411 NCT04379336 NCT04348370 (phase IV)

Trial information is taken from http://www.clinicaltrials.gov. In the case of completed trials, the publication is indicated. Note that this list of trials is not complete.
